# Socioeconomic status influences the relationship between residential green space and the risk of osteoporosis among rural adults: a large-scale population-based study

**DOI:** 10.3389/fpubh.2025.1695153

**Published:** 2026-01-09

**Authors:** Jianwen Li, Hongfei Zhao, Fengling Wang, Chongjian Wang, Jun Pan

**Affiliations:** 1 Department of Orthopaedic Surgery, Yongjia County People's Hospital, Wenzhou, Zhejiang, China; 2 Zhumadian Center for Disease Control and Prevention, Zhumadian, Henan, China; 3 School of Public Health, Gansu University of Chinese Medicine, Lanzhou, Gansu, China; 4 Department of Epidemiology and Biostatistics, College of Public Health, Zhengzhou University, Zhengzhou, Henan, China; 5 Department of Orthopaedic Surgery, The Second Clinical Medical School, The Second Affiliated Hospital and Yuying Children’s Hospital of Wenzhou Medical University, Wenzhou, Zhejiang, China

**Keywords:** health inequity, osteoporosis, residential green space, rural adults, socioeconomic status

## Abstract

**Objective:**

Residential green space has been associated with a range of chronic health outcomes. However, its relationship with osteoporosis remains understudied. This study aimed to investigate the association between residential green space exposure and the prevalence of osteoporosis, and further to examine the combined effects of socioeconomic status (SES) on this relationship.

**Methods:**

This study involved 6,544 participants from the Henan Rural Cohort study. Residential green space was measured via normalized difference vegetation index (NDVI) and enhanced vegetation index (EVI). SES was assessed by marital status, educational level, and average monthly income scores. Logistic regression and restricted cubic spline models were applied to explore the associations between residential green space exposure and osteoporosis risk.

**Results:**

Higher levels of NDVI and EVI were significantly associated with lower odds of osteoporosis. Participants in the middle tertiles (T2) and highest tertiles (T3) of residential green space exposure exhibited lower odds of osteoporosis compared to those in the lowest tertile (T1) [*OR* (95%*CI*), EVI: T2, 1.01 (0.86, 1.18) and T3, 0.63 (0.54, 0.74); NDVI: T2, 0.73 (0.63, 0.86) and T3, 0.60 (0.51, 0.70)]. Higher SES was also associated with lower osteoporosis risk [*OR* (95%*CI*), T2, 0.89 (0.76, 1.03) and T3, 0.78 (0.64, 0.94)]. Moreover, participants with both high greenness exposure and high SES had a significantly lower risk of osteoporosis.

**Conclusion:**

This study indicated that greater exposure to residential green space could be associated with a reduced risk of osteoporosis among rural adults, with SES potentially enhancing this relationship. These findings provide valuable insights for developing public health strategies to prevent and control osteoporosis in rural populations.

**Clinical trial registration:**

http://www.chictr.org.cn/showproj.aspx?proj=11375, Identifier ChiCTR-OOC-15006699

## Highlights

- Focused on rural, resource-limited populations.
- First investigation to examine the association between residential green space and osteoporosis. Socioeconomic status modifies the association of residential green space with osteoporosis.
- Provides evidence to inform prevention and control strategies for osteoporosis.

## 1 Introduction

Residential green space has increasingly been recognized as an important determinant of population health, and studies have indicated its potential to reduce the burden of chronic diseases (1, 2). Availability of green areas in residential settings has been significantly associated with lower risks of various conditions, including cardiovascular disease, respiratory diseases, diabetes, and improved mental health (2–4). However, the majority of existing studies have mainly concentrated on developed regions (5, 6). Urbanization driven by economic growth has led to a pronounced decline in green space, now recognized as a major environmental concern. In resource-limited rural areas, enhancing residential green space may serve as a more cost-effective strategy than traditional medical interventions (7). Therefore, given its low cost and high potential benefits, increasing residential green space should be considered an important strategy to improve public health, especially in economically disadvantaged areas.

Osteoporosis has emerged as a noticeable public health concern due to social development. In a large-scale meta-analysis of osteoporosis, the prevalence was 11.7% and 23.1% in men and women, respectively (8). In China, the prevalence of osteoporosis among individuals aged 40 and older was 5.0% for men and 20.6% for women (9). The treatment costs and osteoporosis-associated complications could impose a substantial burden on both individuals and healthcare systems, especially in developing countries (10). Moreover, the situation in China is expected to worsen in the coming years (11). Thus, it is imperative to implement measures to protect bone health.

Osteoporosis is typically associated with key determinants, such as sex and lifestyle (12, 13), while research on the emerging risk factor of environmental exposure remains limited. Prior research has revealed that a higher level of green space exposure was associated with a slower increase in lumbar spine bone mineral density and a higher risk of fracture (14). Another study demonstrated that living near green space is linked to improved bone strength (15). A prospective cohort study from the UK Biobank indicated that public greenery and natural environments could reduce the risk of developing osteoporosis (16). While higher socioeconomic status (SES) has been identified as a protective factor against osteoporosis, evidence from rural areas remains limited (17, 18). In addition, few studies have explored the combined effects of SES and residential green space exposure on osteoporosis.

To address these gaps, this study aimed to (1) evaluate the association between residential green space exposure and osteoporosis risk and (2) investigate whether SES modifies this association among rural adults from the Henan Rural Cohort Study.

## 2 Materials and methods

### 2.1 Study population

Participants were drawn from the Henan Rural Cohort Study (19), a large ongoing prospective cohort. In brief, this study was designed to investigate the prevalence and influencing factors of chronic metabolic diseases in rural areas. Participants were recruited using a multi-stage stratified cluster sampling method across Henan Province. Five rural areas were included: Suiping County, Yuzhou County, Xinxiang County, Tongxu County, and Yima County. The baseline survey was conducted from 2015 to 2017, and the follow-up survey was conducted from 2018. The bone mineral density ultrasound examination was performed and analyzed in the baseline survey (*n* = 6,544). The investigation was approved by the Ethics Committee of Life Sciences of Zhengzhou University, and all participants completed the informed consent.

### 2.2 Assessment of potential covariates

A structured questionnaire administered by trained staff collected demographic characteristics, lifestyle factors, and medical history. Smoking and drinking statuses were categorized as never, former smoking/drinking (quit), and smoking/drinking. Dietary intake was assessed using a validated Food Frequency Questionnaire (20). Intake of more than 75 g of meat per day was defined as a high-fat diet, and an intake of more than 500 g of vegetables and fruits per day was classified as more vegetable and fruit intake. Physical activity levels were assessed using the International Physical Activity Questionnaire-Short form and categorized as low, moderate, and high. Women were classified as postmenopausal if they reported natural menopause or had undergone a hysterectomy. Moreover, the SES score was derived from marital status, education level, and average monthly income, ranging from 0 to 7. The marital status was divided into two groups: widowed/separated/divorced = 0, married/cohabiting = 1; the education levels were classified into illiteracy = 0, primary = 1, junior high school = 2, senior high school or above = 3; and the income levels were assigned into <500 = 0, 500 ~ = 1, 1,000 ~ = 2, 2,000 ~ = 3.

### 2.3 Assessment of residential greenness

In this study, residential green space included both public green areas and agricultural crops. We assessed green space exposure using the enhanced vegetation index (EVI) and normalized difference vegetation index (NDVI), derived from the Moderate Resolution Imaging Spectroradiometer (MODIS) Terra onboard the TERRA satellite at a 250 m by 250 m resolution (21). We collected the geolocation information of residents’ addresses and created buffers around each point. The 16-day NDVI/EVI data were aggregated by calculating the average value for the 3 years preceding the baseline survey (22). EVI and NDVI values range from −0.2 to +1, with higher values indicating a larger green area. This study primarily focused on the mean EVI [1,000-m buffer radius; EVI (1,000 m)] and NDVI [1,000-m buffer radius; NDVI (1,000 m)] values averaged over 3 years. In addition, results for individual years and for different buffer radii are presented in the Supplementary material.

### 2.4 Assessment of bone mineral density

Bone mineral density (BMD) was measured using a calcaneal quantitative ultrasound device (Hologic Sahara, USA). T-scores were calculated using reference values from healthy young adults. We gained the T score and bone mineral density values and then analyzed the average values. In addition, participants with a T score ≤ −2.5 were defined as having osteoporosis, in accordance with previous studies (23, 24).

### 2.5 Statistical analysis

Baseline characteristics of participants were compared between those with and without osteoporosis, using chi-squared tests and *t*-tests. We investigated the associations of environmental vegetation indices (EVI and NDVI) and SES scores with bone health [bone mineral density (BMD) and osteoporosis risk] via a restricted cubic spline plot. Then these variables (EVI, NDVI and SES scores) were subsequently divided into trisection: the first tertile, the second tertile, the third tertile (T1, T2, and T3), and generalized linear and logistic regression models were employed to analyze the linkage above with BMD and osteoporosis risk, with one model unadjusted and another adjusted for age, sex, health behaviors, BMI, and menopause status (women). The Directed Acyclic Graphs (DAGs) were used for variable selection (Supplementary Figure S1). In addition, the robustness of the current finding was further assessed by performing sensitivity analyses using different averaging periods (1, 2, 4, and 5 years) and different buffer radii for the environmental vegetation indices (EVI and NDVI). Finally, we explored the potential interaction between SES and green space exposure on both BMD levels and osteoporosis risk. Generalized linear models, logistic regression, chi-squared tests, and *t*-tests were conducted using SPSS 24.0. Restricted cubic splines (RCS) were analyzed with SAS 9.1 and visualized in R 4.1.3. All tests were two-tailed, with a significance level of *p* < 0.05.

## 3 Results

### 3.1 Basic characteristics of participants

Table 1 summarizes the baseline characteristics of participants categorized by the presence or absence of osteoporosis. The mean age was 54.21 years (SD 10.76) for participants without osteoporosis and 59.84 years (SD 10.56) for those with osteoporosis. Compared to the healthy control group, participants with osteoporosis showed significantly higher age, proportion of women, smoking status, alcohol consumption status, and menopause status in women. But lower in SES scores, proportion of high fat diet and proportion of more vegetable and fruit intake, BMI and NDVI_1000m_.

**Table 1:** Basic characteristics of study participants according to osteoporosis (OP) (*n* = 6,544).

Variables	Total (*n* = 6,544)	Non-OP (*n* = 5,185)	OP (*n* = 1,359)	*p*
Age (years, mean ± SD)	55.38 ± 10.96	54.21 ± 10.76	59.84 ± 10.56	<0.001^a^
Sex (*n*, %)				<0.001^b^
Female	3,977 (60.77)	3,004 (57.94)	973 (71.60)	
SES scores (mean ± SD)	3.46 ± 1.52	3.57 ± 1.50	3.06 ± 1.53	<0.001^a^
Smoking status (*n*, %)				<0.001^b^
Never	4,813 (73.55)	3,730 (71.94)	1,083 (79.69)	
Former	317 (4.84)	279 (5.38)	38 (2.80)	
Current	1,414 (21.61)	1,176 (22.68)	238 (17.51)	
Drinking status (*n*, %)				<0.001^b^
Never	5,091 (77.80)	3,956 (76.30)	1,135 (83.52)	
Former	917 (14.01)	784 (15.12)	133 (9.79)	
Current	536 (8.19)	445 (8.58)	91 (6.70)	
High-fat diet (*n*, %)	1,288 (19.69)	1,099 (21.20)	189 (13.91)	<0.001^b^
Physical activity (*n*, %)				0.121^b^
Low	1715 (26.21)	1,335 (25.75)	380 (27.96)	
Moderate	2,423 (37.03)	1949 (37.59)	474 (34.88)	
High	2,406 (36.77)	1901 (36.66)	505 (37.16)	
More vegetable and fruit intake (*n*, %)	3,665 (56.01)	3,053 (58.88)	612 (45.03)	<0.001^b^
BMI (mean ± SD)	24.50 ± 3.45	24.70 ± 3.39	23.74 ± 3.57	<0.001^a^
Menopause status in women (*n*, %)	2,575 (65.12)	1759 (58.91)	816 (84.30)	<0.001^b^
BMD (g/cm^2^, mean ± SD)	0.44 ± 0.12	0.47 ± 0.10	0.29 ± 0.04	<0.001^a^
EVI_1000m_ (mean ± SD)	0.371 ± 0.032	0.372 ± 0.032	0.370 ± 0.030	0.073^a^
NDVI_1000m_ (mean ± SD)	0.530 ± 0.032	0.531 ± 0.032	0.529 ± 0.031	0.048^a^

SD, standard deviation; SES, socioeconomic status; BMI, body mass index; BMD, bone mineral density; EVI, enhanced vegetation index; NDVI, normalized difference vegetation index.

a *t*-Test was performed to compare the differences in continuous variables.

b Chi-squared test was used to compare the differences in the categorical variables.

### 3.2 Dose–response relationship of green space levels and SES scores with osteoporosis

**Figure 1:**
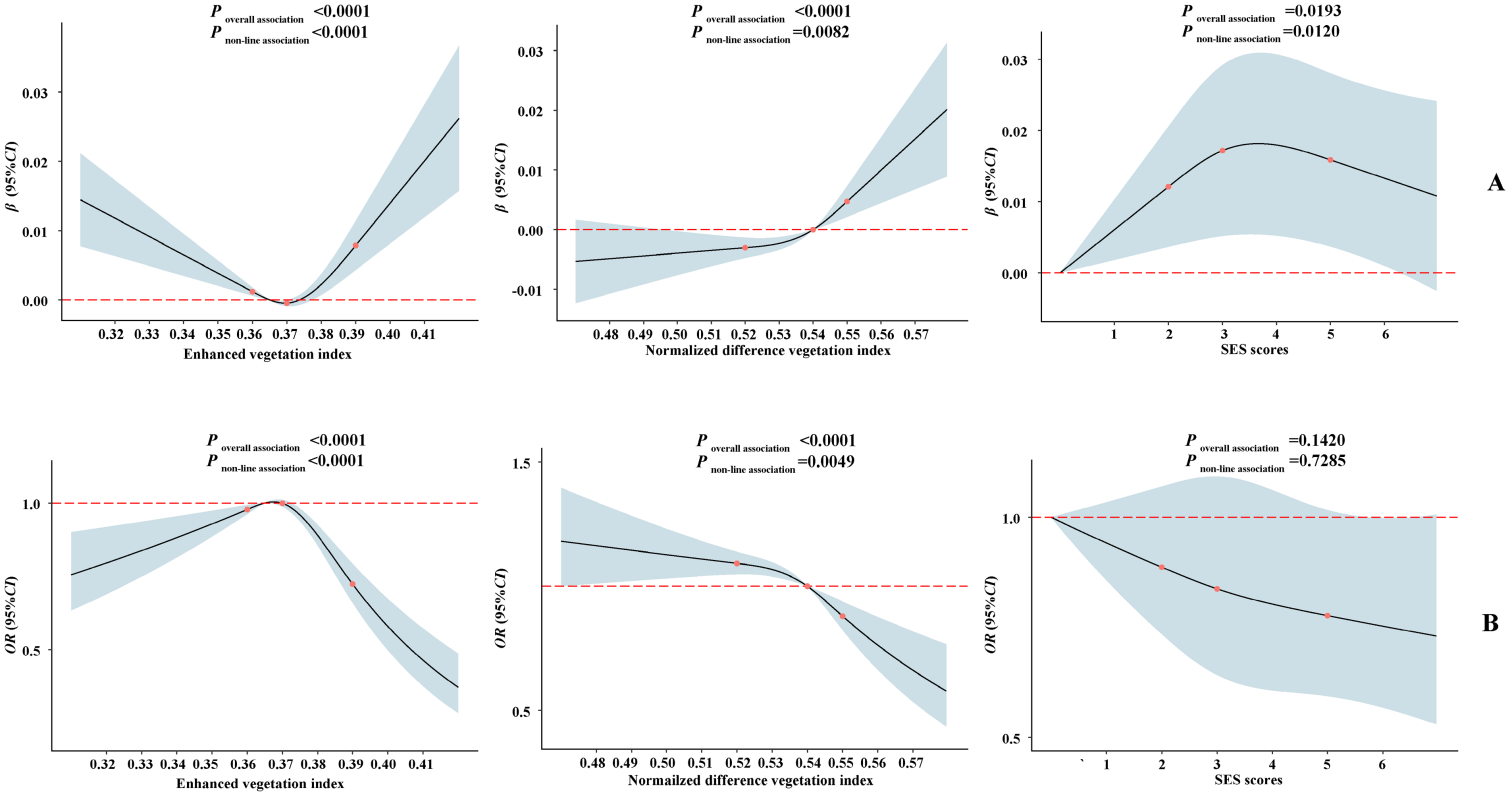
Dose–response relationship of greenness indices and SES scores with BMD **(A)** and osteoporosis risk **(B)**. Adjusted for age, sex, smoking status, drinking status, high-fat diet, more vegetable and fruit intake, physical activity, BMI, and menopause status (women).

Restricted cubic spline analyses were used to explore the dose–response relationships of green space levels and SES scores with bone health. The results showed that BMD augmented with increasing levels of EVI, NDVI, and SES scores (Figure 1A, all *p* for overall association and *p* for non-linearity <0.05). Furthermore, the prevalence of osteoporosis decreased significantly with increasing the levels of EVI, NDVI, and SES scores (Figure 1B). However, the association between SES scores and osteoporosis prevalence was not statistically significant.

### 3.3 Association among different groups of green space levels and SES scores with osteoporosis

**Figure 2:**
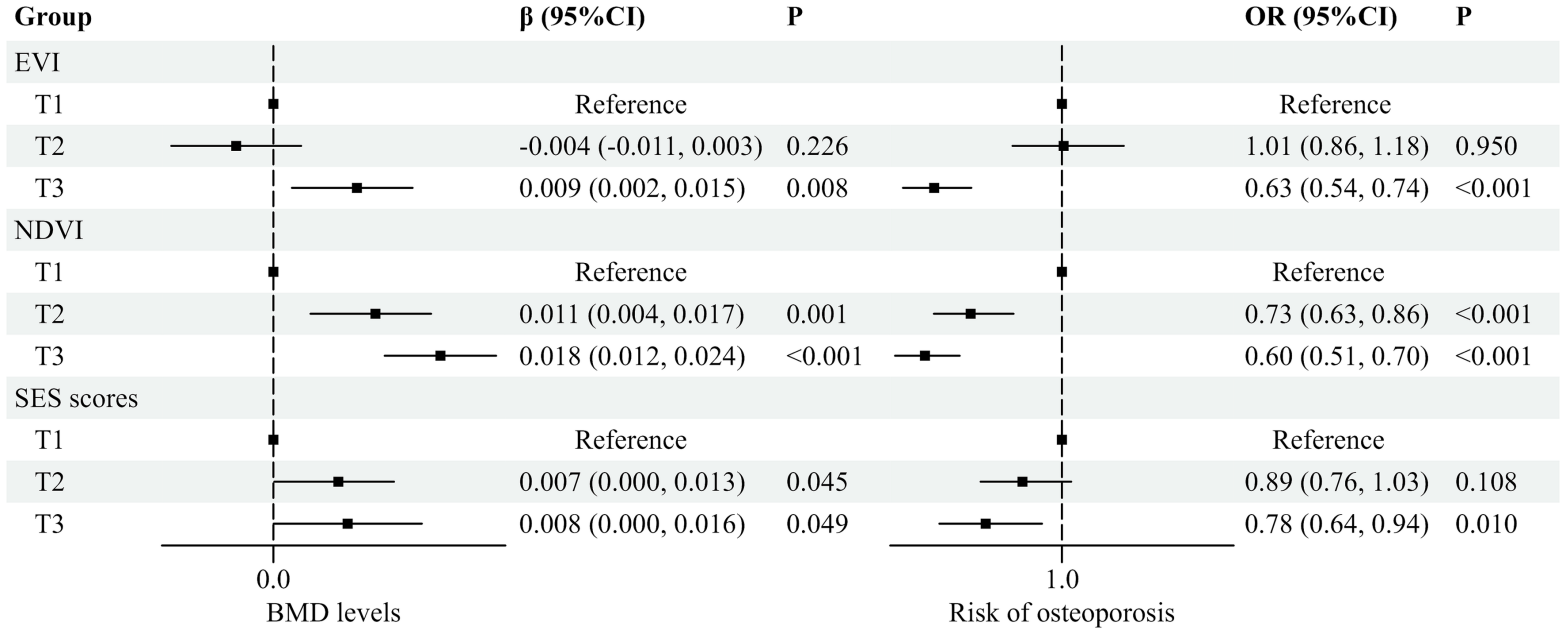
Association among different groups of greenness indices and SES with BMD and osteoporosis risk. Adjusted for age, sex, smoking status, drinking status, high-fat diet, more vegetable and fruit intake, physical activity, BMI, and menopause status (women). The first tertile: T1, the second tertile: T2, the third tertile: T3.

Compared with the subjects in the first tertile, the adjusted *β* (95% *CI*) was 0.009 (0.002, 0.015) in T3 of EVI about BMD. NDVI showed similar results [T2: 0.011 (0.004, 0.017), T3: 0.018 (0.012, 0.024)]. In addition, SES scores might manifest a positive relationship with BMD [T2: 0.007 (0.000, 0.013), T3: 0.008 (0.000, 0.016), relative to the T1], and osteoporosis risk [*OR* (95% *CI*)]: 0.63 (0.54, 0.74) in T3 of EVI, NDVI: T2, 0.73 (0.63, 0.86); T3, 0.60 (0.51, 0.70), and T3: 0.78 (0.64, 0.94) in SES scores (Figure 2). In addition, the associations still remained robust after stratification by sex (Supplementary Tables S1, S2). Supplementary Figure S2 showed that the *OR* (95% *CI*) of the associations between green areas and risk of osteoporosis remained virtually robust in the averages of different years of EVI/NDVI. Moreover, the 3,000-m buffer radius yielded consistent results, whereas the 500-m buffer radius showed different patterns (Supplementary Table S3). The sensitivity analysis was considered with different cut-points and continuous exposure as in Supplementary Table S4, which was similar to the above.

### 3.4 Combined effect analyses

**Figure 3:**
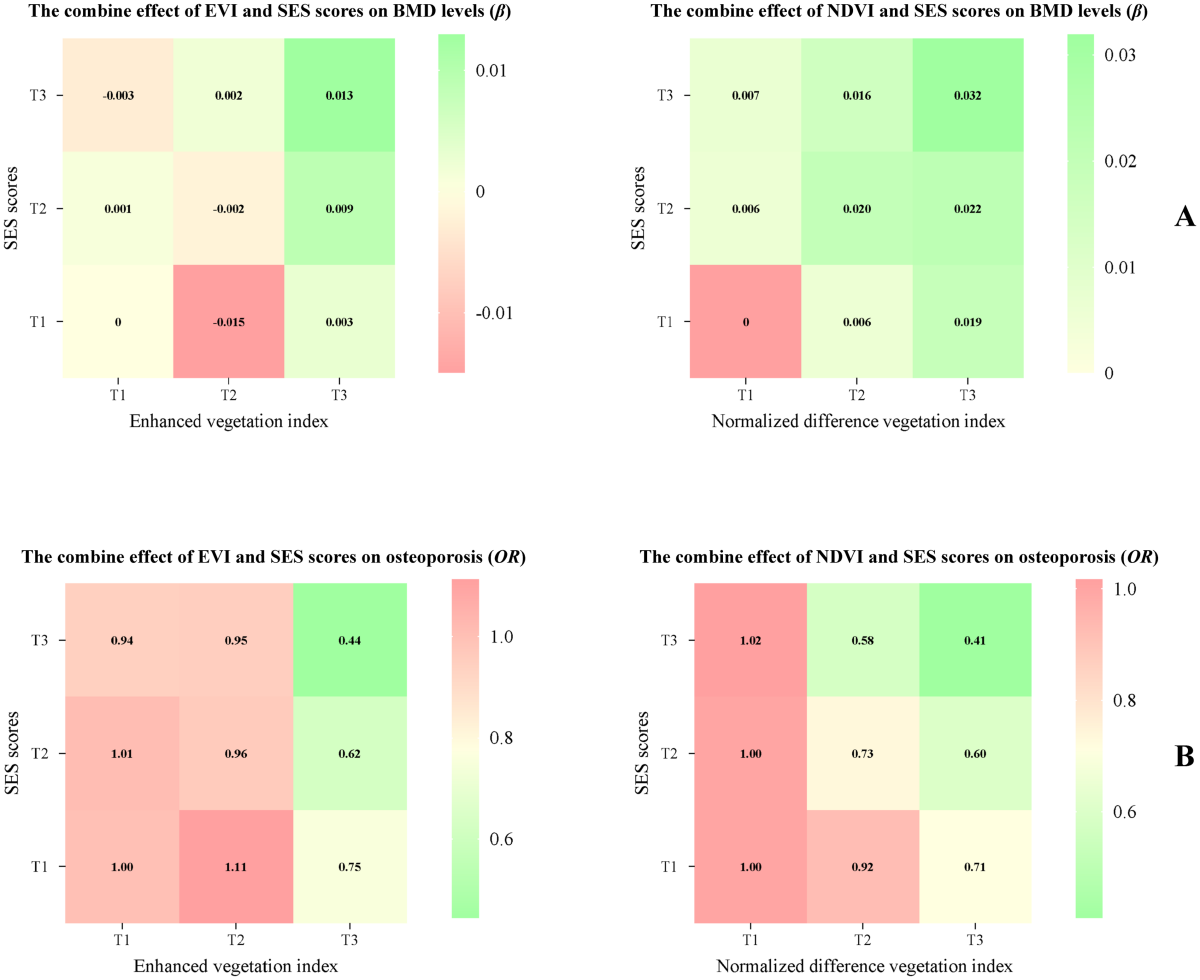
The joint effect of EVI and NDVI with SES scores on BMD levels and osteoporosis. Adjusted for age, sex, smoking status, drinking status, high-fat diet, more vegetable and fruit intake, physical activity, BMI, and menopause status (women). The first tertile: T1, the second tertile: T2, the third tertile: T3.

Figure 3 shows the combined effects of the SES score and green space exposure. Compared to participants with the lowest levels of both SES and green space, subjects with a higher SES score and higher green space exposure had significantly higher BMD (Figure 3A) and lower risk of osteoporosis (Figure 3B). However, this protective association was not observed in participants with a middle level of EVI combined with a high SES score.

## 4 Discussion

This large population-based study provides evidence that residential green space exposure and socioeconomic status are independently and jointly associated with improved bone health among rural adults. Higher exposure to residential vegetation was related to both higher BMD and lower prevalence of osteoporosis. SES modified this association, highlighting the complex interplay between environmental and socioeconomic determinants of health.

A key strength of our study is the use of two validated vegetation indices. NDVI is one of the widely used indicators to evaluate green space, which was used in previous studies (25, 26). EVI enhances vegetation monitoring by incorporating blue wavelengths to mitigate atmospheric interference and minimize soil background noise, offering improved accuracy over NDVI through better separation of canopy signals (27). By employing both indices, we aimed to provide a more robust assessment of greenness, with EVI being particularly suited to the rural, vegetated environment of our cohort. In line with previous studies (28, 29), we used a 1,000-m buffer radius for the analysis, which might better cover the scope of most of the daily activities of the rural population. The most common buffer size in the GIS analysis, generally consistent with a 10-min walk, was 0.5 miles (approximately 800 m) (30), which also fits our choice.

Studies increasingly suggest that living near green spaces is linked to a lower risk of certain diseases. A large-scale meta-analysis confirmed the significant beneficial effects of residential green space exposure on cardiovascular health (1). In addition, increasing residential green spaces can enhance the moderate cognitive benefits of middle-aged women (7). However, limited attention has been paid to the association of bone health and green space levels. Studies investigating the link between green space and bone health have shown mixed results. Higher exposure to residential green spaces has been associated with slower increases in bone mineral density (BMD) and a higher risk of fractures (14, 31). These effects may be influenced by the level of greenness, which can be negatively correlated with certain physical activities, such as walking and cycling (32, 33), that are beneficial for bone health (31). Conversely, some types of physical exercises may increase the risk of fractures (31, 34). This contrasts with a Chinese cross-sectional study, which suggested that green space may be beneficial for bone strength (15). Furthermore, in the UK Biobank study, each quartile increase in exposure to green and natural environments was associated with 4% lower risk of osteoporosis (16), and similar findings have been observed in children (35). In the present research, we found significant associations of the level of residential green space (NDVI with BMD) and risk of osteoporosis. Still, a U-shaped dose–response relationship between residential green space (using EVI) and bone health, which could result from the level of EVI, as a more precise indicator for evaluating green spaces, needs to reach a certain threshold to observe a clear effect (36), which is a hypothesis that warrants further investigation, or the samples with low EVI levels are relatively few so the results are somewhat limited (15).

Building on existing research, our study also examines the association between SES and bone health. A study utilizing National Health and Nutrition Examination Survey (NHANES) data found that higher SES correlated with increased lumbar BMD in US male participants (17). An analysis of the KNHANES data revealed that low SES was significantly linked to a higher prevalence of osteoporosis among Korean menopausal women (37). In a previously understudied population of rural Chinese adults, our findings align with these observations. More importantly, we discovered the combined effect of SES and residential green space on bone health.

While the biological and environmental mechanisms connecting SES and green space exposure to osteoporosis have not been fully elucidated, some potential explanations could account for this association. First, vegetation can absorb air pollutants, thereby improving air quality (35, 36). This is potentially beneficial for bone health, as air pollution is an emerging risk factor for osteoporosis (38–42). Hence, higher residential green space exposure may promote better bone health. Second, green spaces provide more opportunities for physical activity, which helps build bone density and can reduce stress, thereby promoting relaxation (43). Better mental health, in turn, may have a positive effect on bone metabolism (44). Therefore, more exploration of this topic should be encouraged in the future. Furthermore, the effect of SES on bone can be explained by several factors. Individuals with lower SES may be engaged in manual labor that is detrimental to bone quality (17), whereas those with higher SES tend to have more opportunities to exercise (45) and less stress, both of which are favorable for bone metabolism (46). However, key potential mediators were not measured in this study. Therefore, more exploration of this topic should be encouraged in the future.

Although this is the first large sample size study to explore the independent and combined association of residential green space and SES with osteoporosis risk in resource-limited areas, some limitations should be mentioned and considered in the current research. First, causal inferences cannot be drawn from a cross-sectional study, which may limit the reliability of the findings. Therefore, these results require verification in future prospective studies. Second, residential green space was estimated at the community level instead of the individual level, leading to misclassification. However, there is evidence that it is biased in the direction of the null (47). In future investigations, the assessment of the degree of greenness at the individual level will be taken into consideration. Also, the results of different average values in the sensitivity analysis proved the robustness. In addition, we measured the BMD by the ultrasonic bone density apparatus rather than dual-energy X-ray absorptiometry. However, the significant difference between the QUS and X-ray densitometric BMD methods had not been observed in separating normal from osteoporotic subjects by ROC analysis, and the same measurement method was applied in other studies (15, 48). Moreover, the scoring criteria of SES scores only include three indices (education, income, and marital status), and may be subject to bias and error. But we draw on a number of studies, which might increase the current credibility (49–54). In addition, NDVI/EVI at a 1,000-m buffer may not reflect actual access to or use of green space, especially in rural settings where agricultural land may artificially inflate greenness values. However, crops might also play the role of traditional green plants to some extent, and the sensitivity analysis enhanced the reliability of the results. In addition, the key mechanisms of green space toward osteoporosis are most likely due to physical activity. However, physical activity was evaluated using a self-reported measure, which might result in inaccurate results, reflecting the actual participation in the true intensity of physical activity. In future research, wearable technology could be incorporated to obtain more precise and objective physical activity data in large and representative samples of the population and further verify the associations observed using self-reported physical activity measurements (55). Furthermore, it would be necessary to assess how much of the activity is done within green space and how much time is spent there, although an individual may live near green space. Finally, our results were based on the rural population, but this finding is still significant because it afforded direct evidence for the link between green space exposure and the prevalence of osteoporosis.

## 5 Conclusion

Residential green space exposure and socioeconomic status were associated with improved bone health and reduced osteoporosis risk among rural adults. SES appeared to amplify the protective effects of greenness. Notably, SES and the levels of residential green space may have a joint effect on bone health. These findings highlight potential health disparities and emphasize the importance of both SES and access to green spaces for maintaining bone health in rural populations. Greater awareness of the potential beneficial effects of residential green spaces and SES on bone health should be promoted, and the management of residential green spaces should be implemented as a public health intervention, especially in low-resource settings. More importantly, future longitudinal studies in diverse populations are needed to confirm these associations and to explore causal relationships and underlying mechanisms.

## Data Availability

The raw data supporting the conclusions of this article will be made available by the authors, without undue reservation.
