# Supplementary material for: Socioeconomic status influences the relationship between residential green space and the risk of osteoporosis among rural adults: a large-scale population-based study
Source: Front Public Health. 2026 Jan 9;13:1695153. doi: 10.3389/fpubh.2025.1695153 (PMC12827535; doi:10.3389/fpubh.2025.1695153)
Supplement: Supplementary file 1 [file Table_1.docx]

**Supplement Table 1.** Associations between EVI, NDVI and SES scores (according to the number of thirds) with BMD levels by sex

| **Variables** | **Model 1** | **Model 2** |
| --- | --- | --- |
|  | ***β* (95%*CI*)** | ***β* (95%*CI*)** |
| **Men** |  |  |
| EVI |  |  |
| T1 | 0.00 | 0.00 |
| T2 | -0.007 (-0.017, 0.004) | -0.005 (-0.016, 0.006) |
| T3 | 0.005 (-0.004, 0.015) | 0.005 (-0.005, 0.015) |
| NDVI |  |  |
| T1 | 0.00 | 0.00 |
| T2 | **0.013 (0.003, 0.023)** | **0.014 (0.004, 0.024)** |
| T3 | **0.017 (0.008, 0.027)** | **0.020 (0.010, 0.029)** |
| SES scores |  |  |
| T1 | 0.00 | 0.00 |
| T2 | 0.008 (-0.003, 0.018) | 0.005 (-0.003, 0.013) |
| T3 | **0.020 (0.008, 0.032)** | -0.001 (-0.011, 0.009) |
| **Women** |  |  |
| EVI |  |  |
| T1 | 0.00 | 0.00 |
| T2 | **-0.013 (-0.-23, -0.004)** | -0.005 (-0.014, 0.04) |
| T3 | 0.002 (-0.007, 0.011) | **0.011 (0.003, 0.019)** |
| NDVI |  |  |
| T1 | 0.00 | 0.00 |
| T2 | 0.000 (-0.009, 0.09) | 0.008 (-0.001, 0.016) |
| T3 | 0.008 (-0.001, 0.017) | **0.018 (0.010, 0.026)** |
| SES scores |  |  |
| T1 | 0.00 | 0.00 |
| T2 | **0.034 (0.025, 0.042)** | 0.005 (-0.003, 0.013) |
| T3 | **00.41 (0. 032, 0.051)** | 0.000 (-0.010, 0.010) |

The bold means the difference was significant (*P* <0.05).

Model 1 was unadjusted.

Model 2 adjusted for age, smoking status, drinking status, high fat diet, physical activity, more vegetable and fruit intake, BMI, and menopause status (women).

**Supplement Table 2.** Associations between EVI, NDVI and SES scores (according to the number of thirds) with risk of osteoporosis by sex

| **Variables** | **Model 1** | **Model 2** |
| --- | --- | --- |
|  | ***OR* (95%*CI*)** | ***OR* (95%*CI*)** |
| **Men** |  |  |
| EVI |  |  |
| T1 | 1.00 | 1.00 |
| T2 | 1.07 (0.81, 1.40) | 0.97 (0.73, 1.28) |
| T3 | **0.75 (0.58, 0.98)** | **0.70 (0.53, 0.92)** |
| NDVI |  |  |
| T1 | 1.00 | 1.00 |
| T2 | **0.75 (0.57, 0.98)** | **0.68 (0.51, 0.91)** |
| T3 | **0.75 (0.58, 0.97)** | **0.67 (0.51, 0.88)** |
| SES scores |  |  |
| T1 | 1.00 | 1.00 |
| T2 | **0.75 (0.58, 0.98)** | 0.90 (0.75, 1.08) |
| T3 | **0.51 (0.38, 0.70)** | 0.76 (0.54, 1.07) |
| **Women** |  |  |
| EVI |  |  |
| T1 | 1.00 | 1.00 |
| T2 | 1.19 (0.99, 1.43) | 1.03 (0.85, 1.26) |
| T3 | **0.72 (0.62, 0.83)** | **0.60 (0.49, 0.72)** |
| NDVI |  |  |
| T1 | 1.00 | 1.00 |
| T2 | **0.86 (0.72, 1.03)** | **0.77 (0.64, 0.94)** |
| T3 | **0.67 (0.57, 0.80)** | **0.57 (0.47, 0.70)** |
| SES scores |  |  |
| T1 | **1.00** | 1.00 |
| T2 | **0.56 (0.48, 0.66)** | 0.89 (0.75, 1.07) |
| T3 | **0.41 (0.33, 0.51)** | 0.83 (0.65, 1.05) |

The bold means the difference was significant (*P* <0.05).

Model 1 was unadjusted.

Model 2 adjusted for age, smoking status, drinking status, high fat diet, physical activity, more vegetable and fruit intake, BMI, and menopause status (women).

The first tertile: T1, the second tertile: T2, the third tertile: T3.

**Supplement Table 3.** Associations between EVI and NDVI in different buffer radius (according to the number of thirds) with risk of osteoporosis.

| **Variables** | EVI | NDVI |
| --- | --- | --- |
|  | ***OR* (95%*CI*)** | ***OR* (95%*CI*)** |
| 500m |  |  |
| T1 | 1.00 | 1.00 |
| T2 | **1.27 (1.05, 1.53)** | 0.94 (0.77, 1.15) |
| T3 | 0.93 (0.77, 1.12) | 1.05 (0.87, 1.26) |
| 3000m |  |  |
| T1 | 1.00 | 1.00 |
| T2 | 0.91 (0.75, 1.11) | **0.36 (0.29, 0.46)** |
| T3 | **0.60 (0.49, 0.73)** | **0.47 (0.39, 0.56)** |

The bold means the difference was significant (*P* <0.05). Adjusted for age, smoking status, drinking status, high fat diet, physical activity, more vegetable and fruit intake, BMI, and menopause status (women).

The first tertile: T1, the second tertile: T2, the third tertile: T3.

**Supplement Table 4.** Associations between EVI and NDVI with risk of osteoporosis.

|  | **Variables** | EVI_1000m_ | NDVI_1000m_ |
| --- | --- | --- | --- |
|  |  | ***OR* (95%*CI*)** | ***OR* (95%*CI*)** |
| Medians | < Medians | 1.02 (0.82, 1.25) | **1.71 (1.38, 2.11)** |
|  | Medians | 1.00 | 1.00 |
|  | > Medians | **0.67 (0.55 , 0.82)** | 0.92 (0.74, 1.15) |
| Quartiles | Q1 | 1.00 | 1.00 |
|  | Q2 | 0.98 (0.80, 1.21) | **1.55 (1.22, 1.96)** |
|  | Q3 | **0.76 (0.61, 0.93)** | **0.71 (0.56, 0.91)** |
|  | Q4 | **0.55 (0.43, 0.69)** | 0.80 (0.61, 1.04) |
| Continuous variable | | **0.038 (0.003, 0.464)** | **0.007 (0.001, 0.097)** |

The bold means the difference was significant (*P* <0.05). Adjusted for age, smoking status, drinking status, high fat diet, physical activity, more vegetable and fruit intake, BMI, and menopause status (women).


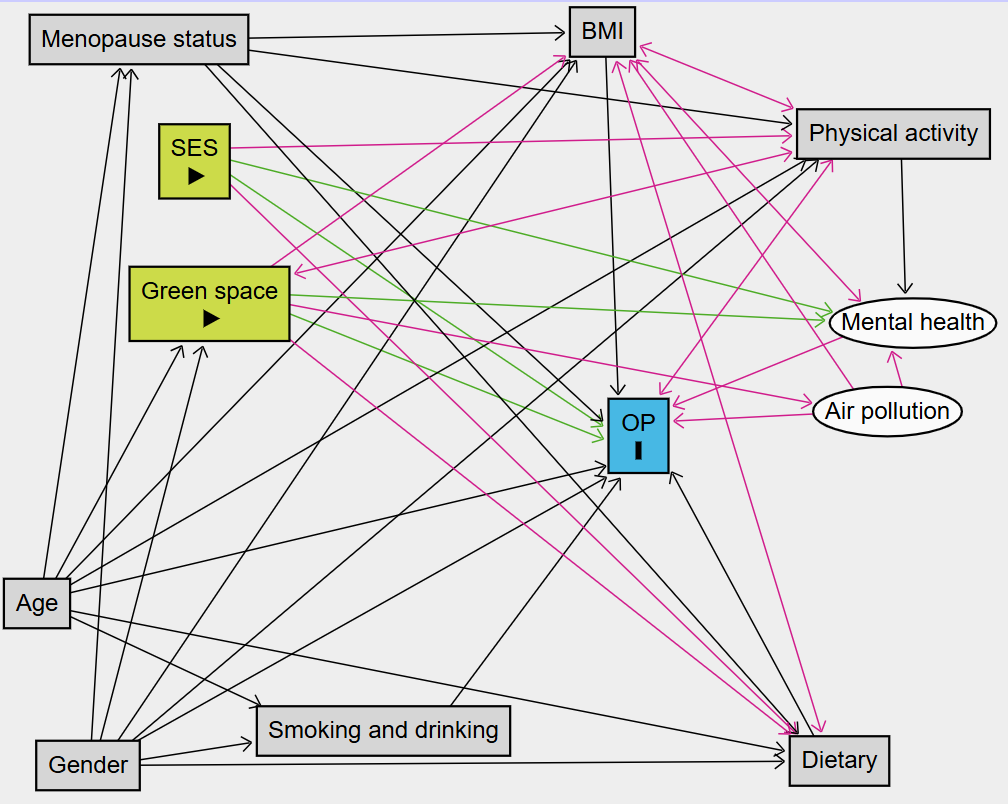
**Supplement Figure 1:** The Directed Acyclic Graphs (DAGs) for variable selection

**
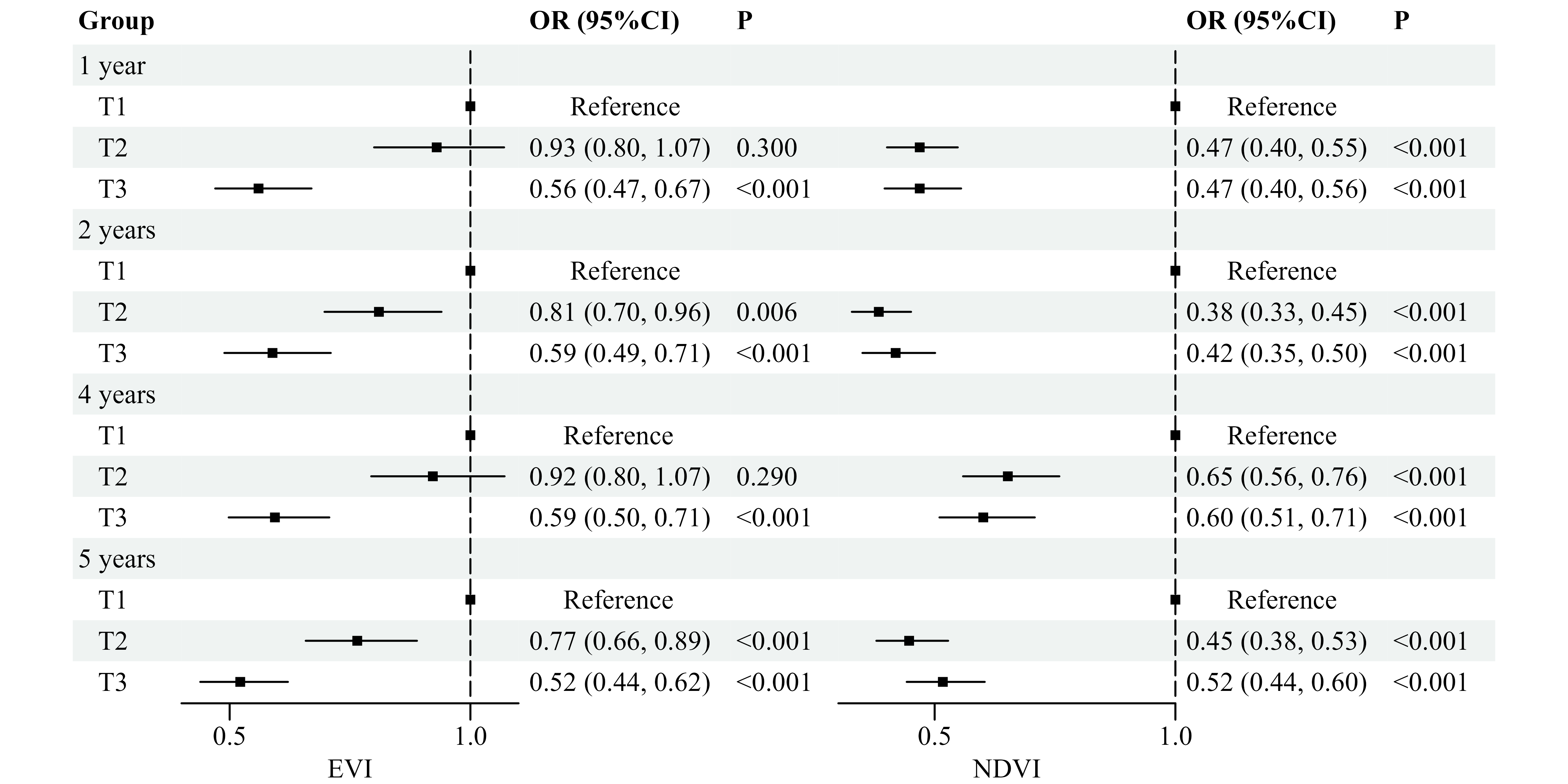
Supplement Figure 2.** Associations between the different years’ averages of EVI and NDVI (according to the number of thirds) with risk of osteoporosis. Adjusted for age, sex, smoking status, drinking status, high fat diet, physical activity, more vegetable and fruit intake, BMI, and menopause status (women).

The first tertile: T1, the second tertile: T2, the third tertile: T3.


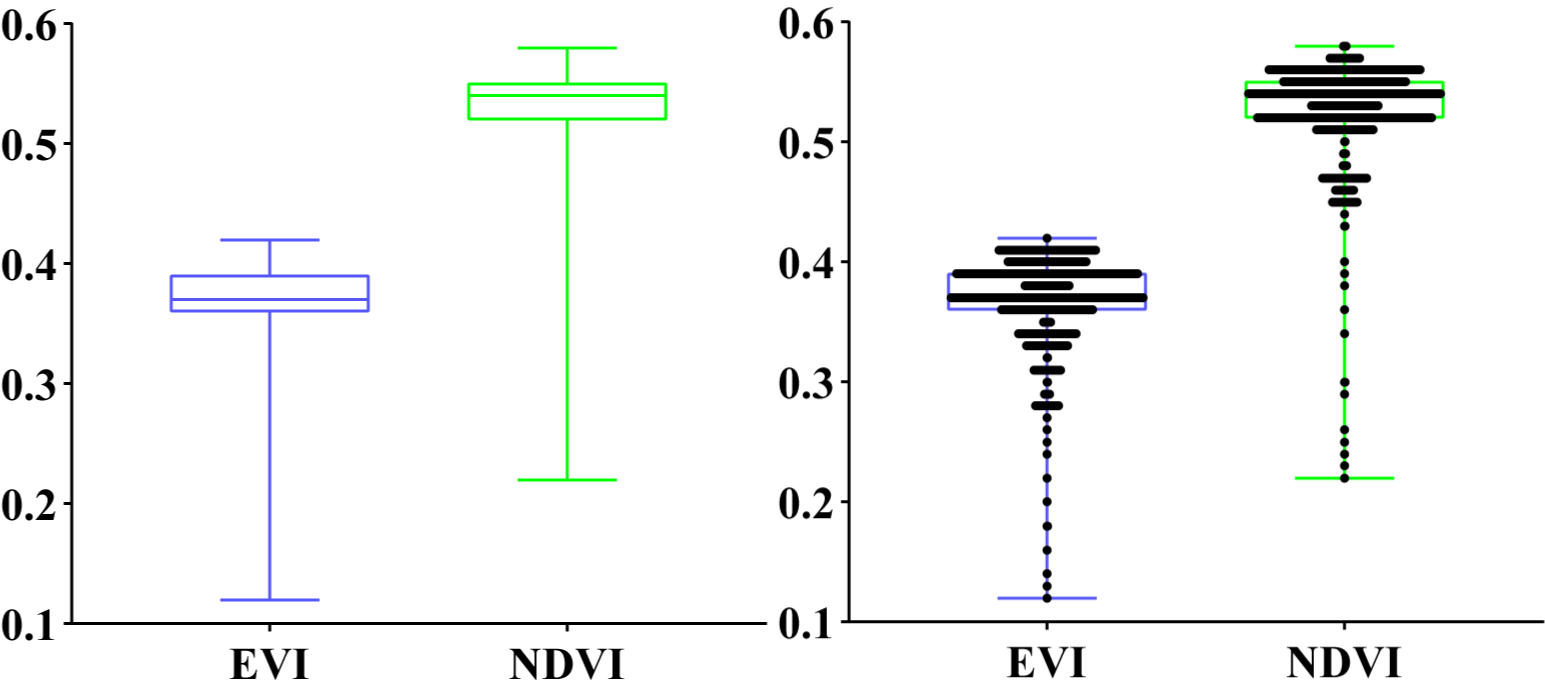


**Supplement Figure 3.** Boxplots of EVI and NDVI.
